# Understanding factors that impact patient access and engagement with biomedical and traditional care for hip fractures in The Gambia: An ethnographic study using a social ecological model

**DOI:** 10.1371/journal.pgph.0006626

**Published:** 2026-07-31

**Authors:** Awa Touray, Rachael Gooberman-Hill, Omar Cessay, Lucy Gates, Rudo M. S. Chingono, Cliff Zinyemba, Jainaba Badjie, Tida Saidy, Kaddy Darboe, Kebba Marenah, Matthew Costa, Celia L. Gregson, Kate A. Ward, Sarah Drew

**Affiliations:** 1 Faculty of Environmental and Life Sciences, School of Health Sciences, University of Southampton, United Kingdom; 2 MRC Unit the Gambia at London School of Hygiene & Tropical Medicine, Fajara, The Gambia; 3 Global Health and Ageing Research Unit, Bristol Medical School, University of Bristol, United Kingdom; 4 School of Healthcare Enterprise and Innovation, Faculty of Medicine, University of Southampton, United Kingdom; 5 The Health Research Unit Zimbabwe, Biomedical Research and Training Institute, Harare, Zimbabwe; 6 Centre on Climate Change and Planetary Health, London School of Hygiene and Tropical Medicine, United Kingdom; 7 Orthopaedic unit, Edward Francis Small Teaching Hospital, The Gambia; 8 Oxford Trauma and Emergency Care, Nuffield Department of Orthopaedics, Rheumatology and Musculoskeletal Sciences, University of Oxford, United Kingdom; 9 MRC Lifecourse Epidemiology, Human Development and Health, University of Southampton, Southampton, United Kingdom; Southern Cross University, AUSTRALIA

## Abstract

Across Africa, the average age of the population is increasing, leading to a rise in health challenges such as hip fractures. Surgical fixation is the standard of care for hip fracture in older adults. In The Gambia, traditional bone setters (TBS) remain an important source of fracture care, though concerns exist regarding complications. In resource-constrained settings, system constraints may also result in post-operative complications. Currently treatment-seeking decisions are poorly understood. We aimed to map hip fracture care pathways to understand factors affecting engagement with TBS and biomedical care. Ethnographic case studies, comprising interviews with 38 patients and 37 caregivers, were complemented by contextual observations of care provision. The social ecological model informed analyses. The model distinguishes five inter-related components influencing illness behaviour: (i) intrapersonal, (ii) interpersonal, (iii) organisational or institutional, (iv) socio-cultural factors, and (v) public policies. Findings showed the complexity of hip fracture care pathways, with most patients moving between TBS, public and private healthcare facilities. Intrapersonal: patients often delayed seeking care because they did not think low-trauma injuries were serious. After visiting TBS, experiences of pain and dependency motivated biomedical care seeking. Some older people avoided surgery because they lacked the “strength” to survive an operation. Interpersonal: decisions about treatment largely rested with caregivers, strongly influenced by finances. Organisational factors favouring TBS included convenience, affordability and prolonged waiting times for operations. Ambiguous terminologies used by healthcare professionals to describe fractures were influential. Socio-cultural factors included beliefs about the causes of fractures, traditions around visiting TBS and fears of biomedical care. Public policy: healthcare financing requiring patients to pay before accessing care encouraged many to seek TBS for timely treatment. Findings highlight the need to co-develop interventions with TBS to help patients navigate complex care pathways and address organisational barriers within biomedical services, whilst respecting local perceptions of TBS.

## Introduction

The Gambia is a West African country undergoing rapid population growth Here life expectancy has risen; between 2013 and 2024, the population aged 65 years and older increased by 25.8% [1]. This rising longevity brings an inevitable increase in the burden of musculoskeletal health conditions such as fragility fractures, including hip fractures [2]. A recent study by Wilson et al. estimated that hip fracture incidence in The Gambia, among adults aged 40 years and older was 28.1 for men and 51.7 for women per 100,000 people. These rates are similar to those reported in Zimbabwe, Botswana, and Black South Africans [3]. Fragility fractures, including hip fractures, occur more commonly in women due to postmenopausal bone loss [4].

Worldwide, surgical management is the biomedical standard of care for hip fracture [5]. Surgery for hip fracture requires metal implants to either fix or replace the broken bone, depending on the fracture type. Before, during and after an operation for hip fracture, hospital-based inpatient care is best delivered within multidisciplinary teams involving nurses, orthopaedic surgeons, physicians and physiotherapists. However, there are significant gaps in provision of inpatient care for hip fractures in The Gambia [6]. Biomedical hip fracture care in The Gambia is intended to be delivered in six stages:

1. Trauma assessment and resuscitation
2. Radiographic imaging to diagnose the hip fracture
3. Pain management and fracture immobilisation, including use of traction in some cases
4. Surgery to fix or replace the broken bone
5. Post-operative rehabilitation
6. Patient health education

Health system challenges, highlighted in the National Health Policy (2021 – 2030), included the rise in non-communicable diseases and shortage of trained healthcare workers [7], with one doctor per 100,000 people [8]. Despite a subsidised health system, patients often encounter high out-of-pocket expenditure at the point of healthcare access [7]. Health care is structured into a three-tier system, linked through a referral system. Tiers comprise: (i) Primary healthcare delivered through village health posts by community healthcare workers who provide promotive and preventive healthcare, (ii) Secondary level care provided through major and minor health centres, including emergency obstetric and neonatal care, and (iii) Tertiary care that comprises four regional hospitals and five General Hospitals; they manage the most complex cases. The Edward Francis Small Teaching Hospital is the only teaching hospital and the main referral hospital [7]

In The Gambia, as well as biomedical care that includes surgery, traditional medicine is a vital and accessible source of care for many people. Traditional medicine refers to the knowledge, skills and practices based on theories, beliefs and experiences within cultures and that are used to maintain health, and to prevent and manage physical and mental illness [9]. In this way, the notion of “medical pluralism” has been used to describe the utilisation of both biomedical and traditional treatment modalities [10]. This pattern of healthcare engagement is widely observed across international contexts and for patients experiencing both chronic and acute. For instance, Pham et al.’s [11] work in Nepal highlighted how patients sought care from both biomedical healthcare professionals and traditional healers, at different points in time, to address both the spiritual and medical causes of mental illness. Likewise, a qualitative study carried out in Uganda identified how patients utilised both traditional and biomedical care throughout the treatment pathway within the pluralistic healthcare system [10]. Traditional bone setters (TBS) are an important part of the traditional medicine system in many countries: TBS are practitioners who treat dislocations, fractures and musculoskeletal injuries based on their knowledge and experience of traditional medicine [12]. Despite their widespread use, Previous research has highlighted serious complications following TBS treatment. In Nigeria, these complications have included malunion and non-union of bones, while in Southern Ethiopia, cases of gangrene have been reported. [13,14]. Furthermore, biomedical treatment including surgery, achieves the best results if treatment takes place soon after the hip fracture, ideally within 48 hours [15]. Previous studies on the management of hip fractures in LMICs have identified that patients who are treated operatively are more likely to survive and have better functional outcomes compared to those who are treated non-operatively [16]. In The Gambia, people with hip fracture do not always attend biomedical healthcare facilities early enough to receive best treatment. Wilson et al. estimated that between July 2022 – July 2024, 30.1% of patients presented over two weeks after injury. Although the reasons for this delay may be complex, visits to TBS are common in the Gambia and may impact on time to presentation in biomedical healthcare facilities. For instance, in a recent study evaluating fracture service availability and readiness in The Gambia, 60% of patients who had presented across 150 health facilities in The Gambia between October 2021 and Jan 2023 had already visited a TBS [6]. In the same study, 42 TBS were identified country-wide, though the absence of a formal register of TBS means that the number is likely to be substantially higher. The ratio of traditional bone setters to the population in The Gambia is therefore unknown. TBS offer services for various musculoskeletal injuries, including sprains, fractures, and muscle injuries. TBS practices vary greatly, with a range of procedures and materials used in the treatment of fractures, including hip fracture. In Nigeria, fractures are often treated with splints made from bamboo, rattan, or palm leaves, tied tightly round the injury with cotton thread or cloth. Injuries are massaged, sometimes alongside the application of herbs. If this treatment is not successful, TBS may also use incantations to aid healing [17].

To understand the internal articulation of healthcare systems, Kleinman suggested that all healthcare systems are composed of three overlapping sectors: the ‘popular’ or lay sector that includes self-care and home remedies; the ‘professional’ sector or institutionalised biomedical care; and the ‘folk’ sector that includes other specialists, including traditional practitioners [18]. Empirical studies have shown that people seeking healthcare do not tend to choose only one of these sectors but rather pursue different therapeutic options at different stages of their care journey [19]. Recent research has used the concept of ‘therapeutic itineraries’ to understand how and why patients navigate different healthcare sectors [19]. This approach highlights how routes through care are shaped by the wider socio-cultural context [20,21].

Several theories can be used to help understand factors that influence treatment-seeking behaviour and therefore how people move between sectors or pursue their therapeutic itineraries [22,23]. These include those largely based on individual decision-making [24] and those that emphasise the role of the wider socio-cultural environment [25,26]. Our study used a socio-ecological model (SEM) since it provides a counter-point to individualistic models such as the Health Belief Model that see individual practices arise from rational decision-making processes [27]. Instead, the SEM highlights the impact of structural factors on behaviour [28]. According to this model, patients’ treatment-seeking behaviour may be influenced by characteristics at five levels: (i) intrapersonal, (ii) interpersonal, (iii) organisational or institutional, (iv) socio-cultural factors and (v) public policies [29].

Globally, the relationship between traditional practitioners and biomedical services is complex. In some healthcare systems and for some conditions there are clear referral processes between traditional practitioners and biomedicine. For instance, in several countries across Asia, Africa and Latin America, traditional birth attendants have been trained to detect obstetric complications during childbirth and refer mothers to appropriate healthcare facilities [30]. However, in other settings including Zambia, traditional birth attendants have been banned due to safety concerns [31]. In The Gambia, TBS and biomedical healthcare sectors have no established interconnecting referral processes. This is similar to other African countries where there has been little or no formal integration of TBS into biomedical care pathways [32]. Patients with hip fractures therefore must negotiate prolonged, fragmented and complex care pathways. There is a lack of empirical evidence documenting the complexity of these care pathways or the factors influencing patients’ decisions to engage with one healthcare sector over the other at different points in the journey. Furthermore, no studies in Africa have explored decisions made by those seeking help for hip fracture. As hip fracture primarily affects older adults, there may be specific considerations that impact how choices are made and what those choices and actions taken are.

This study aimed to: (i) map care pathways for patients with hip fracture in The Gambia, and (ii) understand factors affecting patient access and engagement with TBS or biomedical care throughout the care journey, using the socio-ecological model to inform findings. Insights into why and when patients choose TBS or biomedical care are intended to inform development of interventions that support patient needs within this, and similar, health systems.

## Materials and methods

### Study context

The Gambia is a low-income country in West Africa with a population of 2.42 million (36% rural dwelling) [33]. Annual per capital health expenditure in 2017 was just $25.8USD [7]. The Gambia has well-established private facilities that are concentrated around the capital Banjul; these can either be paid for directly by patients themselves or through health insurance. Healthcare is also provided by a range of non-governmental organisations such as the Medical Research Council. In the National Health Policy (2021 – 2030) which outlines government priorities and strategies for the health sector, drawing on national health system assessments, local studies, WHO guidelines, and regional policies, with the goal of achieving Universal Health Coverage, indicates that communication and coordination across these different providers was identified as a challenge [7].

Since 2018, Edward Francis Small Teaching Hospital, located in Banjul, has operated the only public trauma and orthopaedic service in The Gambia. Two private clinics and one non-governmental organisation provide hip fracture surgery [34]. At the time of data collection there were three qualified Gambian orthopaedic surgeons, along with three non-Gambian surgeons on short term placements; they each operate at Edward Francis Small Teaching Hospital and the private clinics. Between June 2022 – June 2023, 78 patients over 40 years of age presented at Edward Francis Small Teaching Hospital with hip fractures; 43 (59%) were treated surgically.

This work is part of the Fractures-E3 research programme, which investigates the Epidemiology, Ethnography, and Economic impact and care of fractures and musculoskeletal health in The Gambia, South Africa and Zimbabwe. The protocol for this work has been previously published [34].

### Study design

The study used ethnographic case studies [35], comprising interviews with patients and their caregivers and others providing informal care. Interviews were complemented by observations of contexts in which care was given and received. The study is reported broadly in keeping with the items in the Consolidated criteria for reporting qualitative research (COREQ) checklist [36].

### Study setting and population

To facilitate collection of information about a range of treatment-seeking processes and practices the study took place in diverse settings: a Tertiary Referral Centres, a Medical Research Centre, Private Clinics that provide hip fracture care and TBS practices (often their homes). Maximum variation sampling was used to include services with a range of characteristics, taking into account facility type and geography of patient catchment area to include Greater Banjul (urban) and West Kiang (rural) [37].

Potential participants were eligible to take part if they had experienced a hip fracture and were 40 years or older, this was to enable focus on older adults with fragility fractures [38]. Hip fractures were categorised as either “low energy” or “high energy” based on descriptions of the injury mechanism described by patients and their caregivers. “Low energy” fractures or “fragility fractures” are caused by minimal force, such as a fall from standing height or less, and often suggest underlying osteoporosis. In contrast, “high energy” fractures result from significant force such as a car accident [39]. Caregivers such as family members and friends were eligible to take part, in addition to other formal and informal care providers, including neighbours and TBS. Inclusion criteria were adults aged ≥40 years who had sustained a hip fracture and were attending participating health facilities, traditional bone setting or lived locally enough to enable data collection. Individuals were excluded if they were <40 years to focus on patients with fragility fractures. As part of our case study approach, carers and/or family members and other individuals involved in their care aged ≥18 years were also eligible to take part in the study.

Multiple strategies were used to identify potential participants including identification of patients by healthcare professionals working in relevant services, clinical record review, TBS recommendations, and contacting participants who had taken part in other work packages within the Fractures-E3 study who had agreed to further contact. The study team (JB and AT) contacted potential participants by telephone, home visit or in the hospital.

Potential participants were sampled purposively considering age, gender, comorbidities, surgical and TBS treatment [37]. Patients with cognitive impairment were included to ensure equity and representation. The sample comprised case studies constituting 38 individuals with a hip fracture and 37 caregivers. Final sample size was determined by the achievement of information power, being adequate to address the study aims [40]. This sample size was relatively high, shaped by the wide variation in care pathways and the cross-case analysis approach used which involved exploring the similarities and differences between patient experiences. Adequacy of sampling was evaluated throughout using iterative data collection and analysis [40].

### Data collection and recording

Data collection took place between 31^st^ March 2022 and 16^th^ August 2023 and comprised repeated in-depth interviews with people who had hip fracture and their caregivers, complemented by observations of home environments and treatment provision [35].

### Interviews

Single, dyad or triad interviews were conducted with persons who had experienced a hip fracture, along with their caregivers. Topic guides supported data collection and explored pathways through care, experiences and views of treatment and decision-making processes (S1 Appendix) [41]. Depending on a patients’ journey though care, 1 – 2 follow-up interviews were conducted 3 – 5 weeks after the initial interview to understand the evolution of the care pathway. Interviews were carried out by Gambian researchers (AT and JB), whose in-depth understanding of the local context helped them to understand participants’ perspectives. In addition, we carried out repeat data collection helped to build rapport and contributed to the collection of rich data. Interviews lasted 35–60 minutes and were carried out in Mandinka, Wolof or Jola languages, as appropriate. Of a total of 73 interviews, 9 were single interviews, 17 were dyads and 12 triads.

### Observations

Observation carried out in patients’ homes described settings, including adaptations to the environment, activities taking place including caregiving activities and interactions between family members and the people around them (S2 Appendix and S3 Appendix). Observation checklists were used flexibility to guide data collection and fieldnotes were written in English to record observations, as well as an additional material potentially of relevance to study aims, such as use of medications or walking aids and adaptations. Approximately 41 hours of observations were carried out.

### Data analysis

Interviews were audio recorded, translated and transcribed into English to enable researchers in the UK to contribute to data analysis and interpretation. Observations were also written up into full fieldnotes. Analysis was iterative and ongoing and informed further data collection [42]. Analytical memos or short reflective notes were recorded by members of the study team in fieldwork diaries. These summarised themes identified and outlined areas for further enquiry. The research team comprised Gambian (OC, TS, KD and AT), Zimbabwean (RC) and UK-based researchers (SD). Regular, collaborative team meetings were held to review findings and ensure the interpretation of data was grounded in the local context. As part of these meetings, researchers discussed how their professional and personal backgrounds may influence the interpretation of data. [42]. A Framework Approach was conducted in five stages: (i) Familiarisation with the data: a sample of transcripts, observation fieldnotes and analytical memos was reviewed collaboratively by members of the Gambian study team (OC, TS, KD) and the UK team SD.. (ii) Development of a working analytical framework: The team met to develop a preliminary coding framework. This involved identifying codes related to treatment-seeking behaviour. These codes were then transposed onto the five levels of the socio-ecological model [29]. (iii) Applying the analytical framework: Data were imported into NVivo qualitative analysis software. A member of the study team (OC) indexed data using the analytical framework. (iv) Charting data into the framework matrix: Two researchers (TS and KD) used NVivo to summarise excerpts from the data related to the codes. (v) Interpretation: Data mapped onto charts was used to identify patterns in the data and interpret findings. When team members identified data that did not ‘fit’ within the existing analytical framework, they made a note of it. These instances were discussed during weekly meetings, where insights were shared and discussed and used to refine the framework.

### Ethical approval

Ethical approval was provided by The MRC The Gambian Scientific Coordinating Committee (SCC 22975). The Ministry of Health Services and Gambian Government provided approval for the research in public healthcare facilities. All participants were asked to sign, mark a thumb-print a written consent form before data collection. Participants could provide additional consent for the researcher to take photographs. Capacity to consent was assessed by trained research non-clinical personnel, in consultation with clinical colleagues where needed. Participants were deemed unable to provide consent if they were unable to understand and retain information about the research, weigh-up the risks and benefits of participation, or clearly communicate their decision to take part [43]. Where participants lacked decisional capacity, consultees were approached to provide assent using the same processes. Two people with hip fracture were assented through consultees.

## Results

Of 38 people who had fracture, 16 (42.1%) were recruited from an urban Tertiary Referral Hospital, one from a rural medical research centre, 8(2.1%) from private facilities and 13(34.2%) through TBS (Table 1). Caregivers were all family members and included wives, children and grandchildren. Of these, 27(72.9%) were female and 10(27.1%) were male (Table 2). Categorisation was carried out by non-clinical trained researchers; 31(81.5%) were classified as having “low energy” fractures. For ease of reading, we refer to people who had a hip fracture as ‘patients’, but acknowledge this is imprecise since the study incorporates material about journeys into and through different sectors of healthcare, including points in time when people were not patients.

**Table 1:** Characteristics of patients who had experienced a hip fracture (aggregated to ensure anonymity).

Patient characteristics		Total N = 38 N (%)
Gender	Female	27 (71.1)
	Male	11 (28.9)
Age (years)	40-49	3 (7.9)
	50-59	2 (5.3)
	60-69	12 (31.6)
	70-79	9 (23.7)
	80 and above	12 (31.6)
Occupation before hip fracture	Teacher	1(2.6)
	Famer	9(23.7)
	Builder	1(2.6)
	Fisherman	1(2.6)
	Street vendor	3(7.9)
	Market vendor	3(7.9)
	religious leader (Imam)	2(5.3)
	Insurance officer	1(2.6)
	Cleaner	1(2.6)
	Waiter	2(5.3)
	Unemployed	14(36.8)
Mechanism of injury	Low energy	31 (81.6)
	High energy	7 (18.4)
Form of healthcare accessed	Surgery only	4 (10.5)
	Surgery and TBS	12 (31.6)
	TBS only	21 (55.2)
	Still to be operated at end of study	1 (2.6)
Co-morbidities	Hypertension	8 (21.1)
	Diabetes	1 (2.6)
	Cognitive impairment	2 (5.3)
	Other[Asthma, hearing impairment, visual impairment]:	7 (18.4)
	No reported co-morbidities	15 (39.5)
Multimorbidity	Individuals with 2 or more co-morbidities	5 (13.1)

**Table 2:** Characteristics of caregivers or caregivers.

Caregiver/Caregiver Characteristics		Total N = 37 N (%)
Gender	Female	27 (72.9)
	Male	10 (27.0)
Age (years)	20-29	4 (10.8)
	30-39	6 (16.2)
	40-49	9 (24.3)
	50-59	10 (27.0)
	60-69	3 (8.1)
	70-79	2 (5.4)
	Don’t know	3 (8.1)
Relationship to patient	Wife	3 (8.1)
	Daughter	16 (43.2)
	Brother	1 (2.7)
	Son	7 (18.9)
	Granddaughter	2 (5.4)
	Grandson	1 (2.7)
	Daughter-in-law	2 (5.4)
	Sister-in-law	3 (8.1)
	Son-in-law	1 (2.7)
	Niece	1 (2.7)

### Characterising hip fracture care pathways

**Fig 1:**
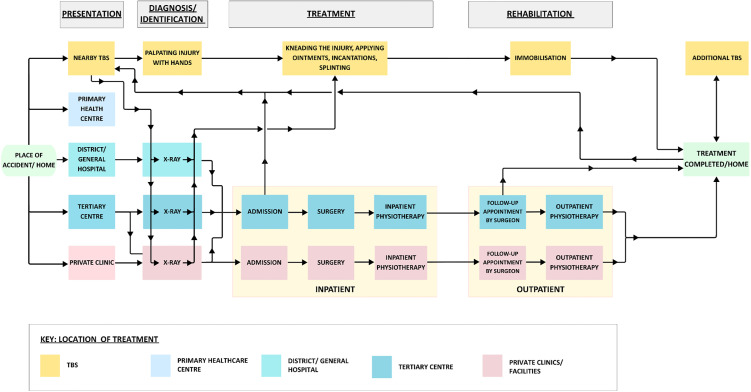
Common pathways through care for hip fracture patients in The Gambia.

Patient and caregiver narratives were used to characterise common care pathways for hip fractures in The Gambia. Patients sought both biomedical care and TBS treatment at different stages of the pathway, including initial presentation, diagnosis, treatment and rehabilitation. Pathways were often non-linear, with patients revisiting different healthcare sectors. Fig 1 illustrates the main routes through care based on patient and caregiver accounts.

**Fig 2:**
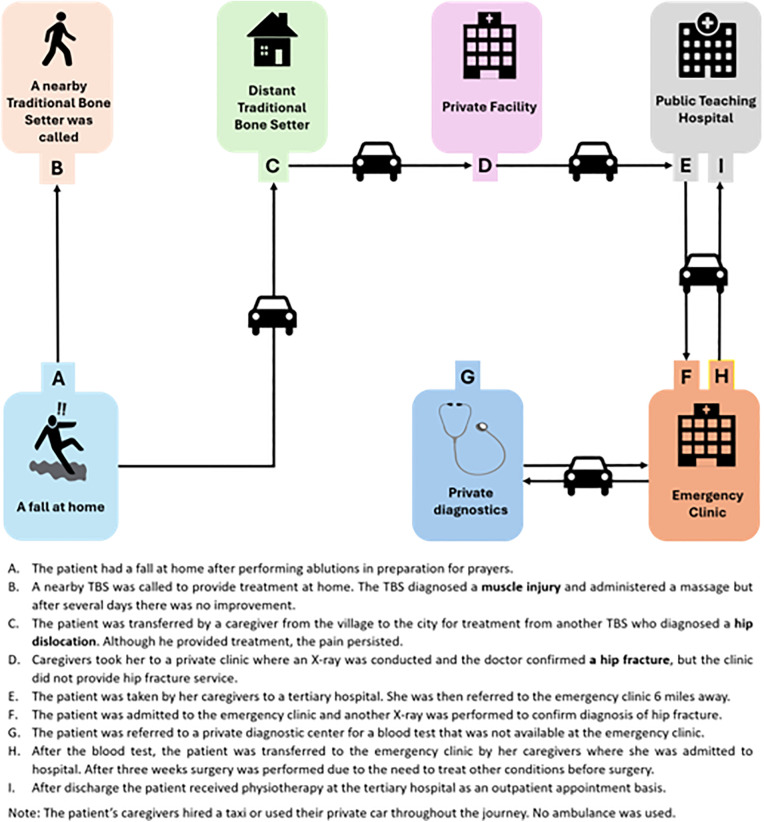
Care pathway for an 85 year-old female patient.

There was wide variation in pathways through care. Fig 2 illustrates the complex route through care for an 85-year-old female patient who fractured her hip following a fall at home.

### Factors impacting access to and engagement with TBS or biomedical care

**Fig 3:**
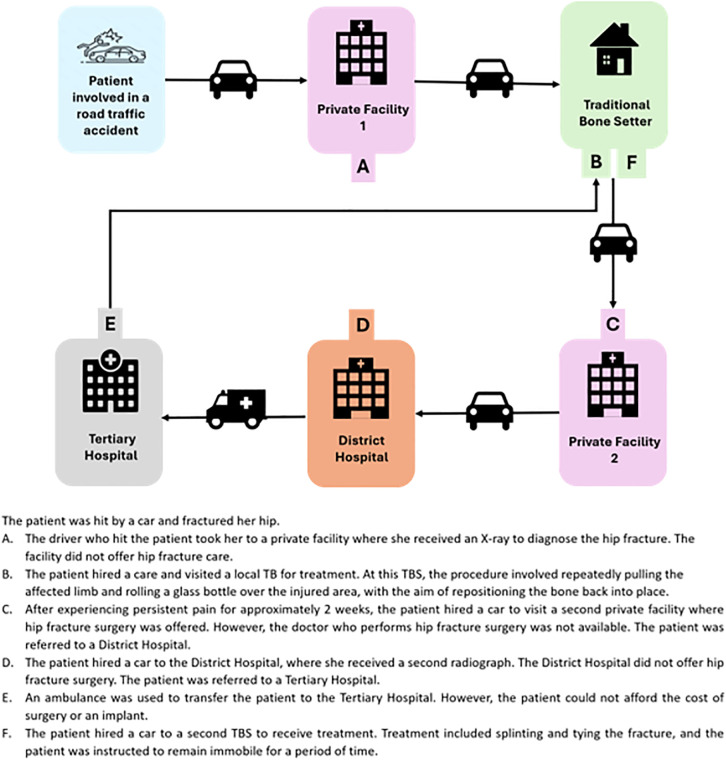
Care pathway for a 55-year-old female patient.

Factors influencing patient access and engagement with biomedical care or TBS treatment were identified at all five levels of the socio-ecological model: (i) intrapersonal, (ii) interpersonal, (iii) organisational or institutional, (iv) socio-cultural, and (v) public policies. These factors were present throughout the care pathway [29]. Fig 3 summarises the main factors influencing treatment-seeking and their relation to the five levels of the model.

#### Intrapersonal factors: ‘Individual characteristics and decision-making’.

Several factors, including injury mechanisms, pain experiences, symptoms and age influenced treatment-seeking. Several patients who described how they had experienced a hip fracture due to low-energy trauma, for instance a fracture that took place when getting out of bed, explained how and why they had delayed seeking care. During observations, patients showed researchers the over-the-counter oral pain relief medications and menthol-based creams they had used to self-manage the fracture rather than visiting TBS or healthcare facilities. These were available from local pharmacies. During observations they demonstrated how they had used shea butter to rub on the painful area that they bought for around 10–50 dalasi (<$1USD). Some patients explained they used shea butter to relieve the pain or heal the injury. Patients and caregivers explained that they visited nearby TBS rather than go to hospital immediately after the fracture because they did not think an injury caused by so little trauma could be serious.

Patients who had been treated by a TBS often expressed that they were in significant pain. During observations, these patients were often seen confined to their chairs and had to be assisted by caregivers to get out of bed or chair with food prepared and brought to them. Caregivers showed researchers the adaptations they had made to their homes, such as moving their mattresses onto the floor so they did not have to climb out of bed and putting a bucket close by so they could assist them with toileting. One 90-year-old female patient who had visited a TBS explained she was now confined to one room in the house. Several patients explained that they now wanted to visit the hospital for pain relief and so they could walk again and regain their independence. By contrast, several older patients and their caregivers explained that they had decided not to have surgery as they were concerned they would not have enough “strength” to survive an operation or that their bodies could not “support” the weight of a metal implant that was too heavy for them (Box 1).

**Box 1: Illustrative quotations - Intrapersonal factors**

*“I didn’t take it seriously to that extent, I never thought it was a fracture initially. I thought it was the way I fell that was why the pain was still there, but it continued so I said to myself, ‘this must not only be a pain [from falling] it must be something else’… I was massaging it using deep heat [heat rub] before going to the hospital.”* Male patient, 65 years old

*“You know, my mother doesn’t have that strength for the metal implant now, she is not that strong. If a younger person is operated on and has a metal implant, the young person will be able to go on his or her business but if an older person has a metal implant, then she will not be able to move.* Daughter and caregiver, 59 years old

#### Interpersonal factors: ‘Interpersonal processes or personal interactions with others’.

During interviews, patients and caregivers explained that decisions about when and where patients sought treatment largely rested with caregivers. Most older patients said that they depended on caregivers for financial and social support and explained that they were often “taken for treatment” by caregivers. Patients and caregivers (both male and female) relayed in interviews how decisions about treatment were often made by the first-born, the most educated, the main breadwinner, or those funding the treatment. A number of patients said that they thought their caregivers had not appreciated the severity of their injury or how painful it was. As such, patients explained how caregivers had encouraged self-treatment or wanted to wait to see if the injury resolved itself before seeking care. One patient described how she had “begged” her family to take her to the hospital due to pain, but they had opted not to take her. Family members explained they later regretted underestimating the seriousness of the injury, believing it was not a major issue.

During interviews, patients and caregivers explained how family financial resources influenced when and where patients sought care. Many families explained that they chose TBS over hospitals due to affordability. To raise funds, they described how they relied on contributions from the extended family, especially from those working abroad. Likewise, in interviews patients explained that they were reliant on practical support from caregivers to enable them to access hospital care. During interviews, caregivers explained how they arranged transport to hospitals or more distant TBS. This would involve either hiring a taxi or using a relative’s car. Supplies of blood in The Gambia are limited due to the small number of blood banks located at tertiary hospitals and lack of willing donors [44]. As such, caregivers described how they located suitable blood donors for operations. They explained how this involved contacting a family member to come to the hospital or reaching out to security services like the fire and rescue services or the army to arrange for blood donation prior to surgery. To enable patients to access biomedical care, female caregivers were observed sleeping on mats outside the hospital so they could help wash and care for the patient during inpatient stays and bought food for patients from vendors outside the hospital. Patients and caregivers also explained how the wider community had also helped with arranging transport and had made small financial contributions when they visited patients in hospital.

Patients and caregivers explained during interviews that treatment decisions were influenced by advice from the extended family and members of the community, who often favoured TBS. Patients and families described how the extended family and community had spoken to them about the benefits of visiting TBS and cited examples of people who had been successfully treated for other types of fractures. Patients explained that caregivers and members of the community made recommendations for specific bone setters based on their personal experiences and the relationships that they had built with them over time. Some had suggested visiting TBS who were widely known, and who they had heard about from friends and family. If treatment from one TBS was unsuccessful, patients explained they had been encouraged by family and friends to visit other TBS in The Gambia or in neighbouring Senegal (Box 2).

**Box 2: *Illustrative quotations - Interpersonal factors***

*“After a while, I observed the pain did not subside it kept increasing, and I found it difficult to walk. I kept receiving recommendations from one person to another to visit a TBS in [Name of the village] and [another village].”* Male patient, 40 years old

*You know the old woman cannot get that money. The family contributes to help, the children and the grandchildren.”* Daughter and caregiver, 45 years old

#### Institutional factors: ‘The processes embedded in organisations’.

Patients and caregivers explained that treatment decisions were strongly influenced by the convenience of access to TBS. Many patients and caregivers said that they had consulted a TBS who lived nearby immediately after the injury, and several described how this TBS had visited their home. They explained that this had saved them transport costs and painful hospital journeys. Both patients and caregivers felt TBS treatment was a faster way of treating fractures than surgery, which often involved long stays in hospital. Data from the interviews highlighted that TBS treatments were provided with a promise of shorter healing times than treatments provided by biomedicine. Due to the ubiquity of TBS throughout The Gambia, many patients discussed visiting multiple TBS if initial treatments failed, delaying hospital visits. Ultimately, perceived failure of TBS treatment and lack of pain relief led patients to seek hospital care.

Families explained they had found TBS cost less to access than hospital care and that they offered flexible payment options, such as deferred payment or costs defined according to ability to pay. In contrast, patients visiting biomedical public facilities described how they faced high costs at each care stage, including payments for operations, prescription costs, payments for beds, and a cost of surgery, including purchasing of implants and sometimes compensation for the blood donor. During observations in hospital, doctors were seen prescribing medications which caregivers were asked to collect from the pharmacy. However, since most of these medications were not available, caregivers explained they were required to source and pay for medications from pharmacies outside of the hospital. During interviews, patients voiced their frustration at needing to do this daily and worried about the accumulation of costs that exceeded their initial expectations.

**Fig 4:**
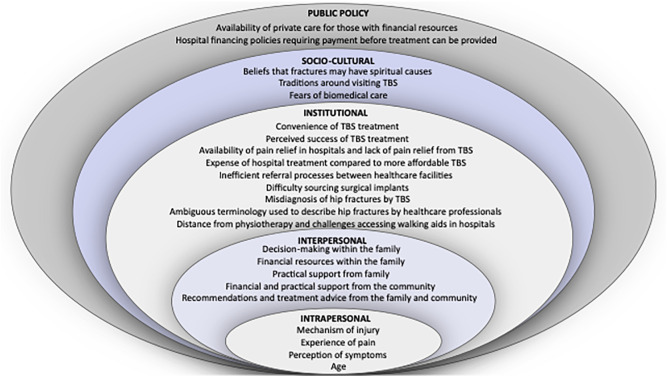
Factors influencing treatment-seeking: codes identified and their relation to the five levels of the social-ecological model.

Based on the costs of treatment that patients reported during interviews, those with the means to access private healthcare incurred higher costs overall. Patients accessing private care explained payments were made either through insurance or through a single invoice before treatment, avoiding stage-by-stage payments. Those who accessed public care explained that the high cost of surgical implants had forced them to abandon biomedical treatment. During interviews, one caregiver showed researchers a written quote of 25,000 Dalasi ($352USD) she had received from a public hospital for an implant (Fig 4). There were no observed differences between public and private hospitals in the cost of implants; patients could pay up to D25,000, depending on the type of implant. There were however differences in surgical costs, as some private patients reported paying up to D50,000 (699.61USD) for surgery. Public hospital patients reported that they were required to pay user fees for consultations, treatments, admissions, and other procedures. [45] Patients and caregivers explained that when costs exceeded available resources, families discontinued hospital care and returned to TBS.

**Fig 5:**
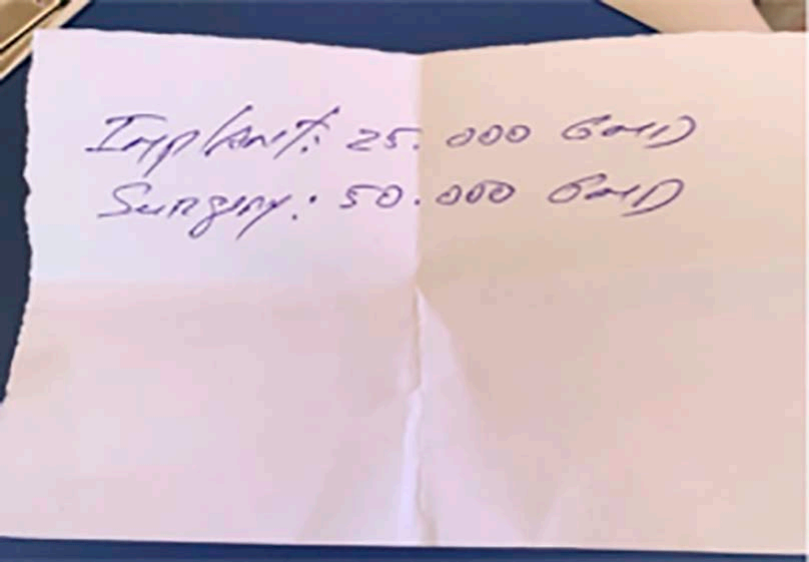
Quote given to a patient by a doctor showing the cost of implant and the fee for surgery.

The quotes for implants and surgical fees are shown in Dalasi (Fig 5). The quote for an implant is 25,000 Dalasi ($352 USD). The quote for surgery is 50,000 Dalasi ($699.61 USD). While recent estimates indicate that a substantial proportion of the Gambian population continues to live below the international poverty line [45].

Patients explained that they faced several organisational barriers in hospitals that had an impact on where they sought treatment. These included Those with fragility fractures explained that they had experienced long waits in orthopaedic clinics at public hospitals before diagnosis and often had to visit multiple public and private facilities before receiving surgical treatment. During interviews, patients and caregivers explained that the limited availability of X-rays at public facilities meant that they had been advised to visit private hospitals or imaging facilities for imaging and diagnosis. A small number of patients who started their journey from a different facility explained they were also required to pay for a repeat e X-ray at the tertiary referral hospital. Where the facility’s X-ray was not functional, patients explained that they were asked to get an X-ray from another hospital or diagnostic facility due to healthcare professionals disregarding the first image. When asked, patients said they believed that this was because healthcare professionals at the tertiary hospital wanted to confirm diagnosis.

Some patients and families explained these challenges had dissuaded them from continuing biomedical care. During interviews, a small number of patients described how they had initially presented at primary health centres or regional and district hospitals and were advised by healthcare professionals to visit a tertiary centre for treatment. These patients explained they were unclear how and where to access these services and subsequently dropped out of biomedical treatment. In care pathways and observations, patients with high energy fractures such as road traffic accidents received quicker hospital treatment due to ambulance transfers prioritising care. During interviews, most private patients, despite some surgery waits, described how they only visited one healthcare facility for treatment

Interview and observational data showed how diagnosis influenced treatment decisions. Some patients who had been told by a TBS that “kulo botaleh” (“the bone is dislocated” in Mandinka) were advised by them to get an x-ray to confirm diagnosis. A small number of these patients explained that they had decided to seek hospital care after learning their injury was a fracture. Interviews demonstrated how the terminology used by healthcare professionals also impacted decisions. Healthcare professionals were observed using several different terminologies to describe hip fractures to patients including “katitaleh” (“broken” in Mandika) and “tayei-taleh” (“cracked” or “broken” in Mandinka). During interviews, a small number of patients and families who were told they had a “cracked bone” (in English) did not think that surgery was needed as they felt the injury did not sound serious. Interviews showed how communication with staff also affected choices. Some patients explained that they did not understand the benefits of surgery or implants, leading them to opt out of hospital treatment. During discharge discussions it was observed that several patients and their families were not provided with information about physiotherapy and rehabilitation. As such, when they were visited in their homes post-discharge, they explained that they were not aware of the service and had not accessed this component of care.

Several patients and their families explained that they had chosen to revisit TBS due to long waits for operations. Reasons provided for this included the need to raise funds for implants, limited availability of operating time, and the need to treat comorbidities. Caregivers discussed the challenges in sourcing implants, as they often needed to be imported from abroad and then paid for through a bank transfer or bought externally from vendors outside the hospital. During rehabilitation, patients and caregivers explained that the distance to facilities limited access to physiotherapy as there was only one public clinic located in a tertiary hospital that provided the service and this had with limited capacity. When patients had limited funds, several explained that they prioritised follow-up appointments with surgeons as they thought these were more important. Hip fracture care in The Gambia is available through both public and private facilities. Patients who sought care in private facilities explained how they received an upfront payment request before the start of treatment that covered all elements of care, including surgery, implants, rehabilitation and all medications. Those who sought care in public healthcare facilities explained how they were asked to pay for their care at the point of access. They described how payment was made sequentially for different components of treatment. Overall, the costs that patients described to researchers were lower in public healthcare facilities than in private care.

Some patients and caregivers explained they were reluctant to attend physiotherapy appointments as they were worried it might be painful. Access to walking aids was inconsistent. Patients and their families often said they were unclear how to access hospital mobility aids. Some patients mentioned that healthcare professionals referred them to the social welfare department where they could access walking aids, while several others were not referred. Patients and their families thought new shop-bought aids were expensive and struggled to locate stores that sold them. As a result, some patients described how they obtained local aids from second-hand shops but were often unsure if they were appropriate for their needs. Others described how they received assistance from TBS in finding aids. Some families also demonstrated how they had improvised with tree branches (Box 3).

**Box 3: Illustrative quotations - Institutional factors**

The quotation below shows how the ambiguous terminology used by healthcare professionals meant that this family did not think that surgery was needed:

*“Money is the problem, they [the hospital] said should operate on him and put metal in the hip, they requested D21,*000 [laughing*] which is difficult to get. Then we took him to a bone setter, and they [the hospital] did the X-ray and told us that it is a “cracked bone”. Imagine, the bone is not broken then how will you put metal in it? Now they are saying D21,000. Where will we get that from? We then took him to a bone setter at [a town]. He should be coming today to see [the patient]”.* Wife and caregiver to patient, 29 years old.

*“They [the healthcare workers at primary healthcare facility] only told us that they don’t treat this type of fracture. We will be the ones to seek who should help the fracture. This is why we went to the bone setter at [name of a village]. This is because they [health workers] don’t treat it so it means we should find a place or someone to treat him”*. Grandson and caregiver to patient, 23 years old.

#### Socio-cultural or community-level factors: ‘Community-level factors such as values and beliefs in the wider society’.

Socio-cultural factors influencing treatment included beliefs about causation, traditions around TBS practices, and fears of biomedical care. Some patients and families said that evil spirits caused fractures and that this meant that certain TBS, who used incantations and supernatural powers, were best suited to heal them. This view was reinforced by some TBS, who were also “marabouts” (traditional healers). A key reason for visiting TBS was the community’s deep faith and familiarity with them. Patients and their families felt that visiting TBS was a way of respecting their ancestors. A small number explained that hospital care for fractures had only recently been introduced in The Gambia and that they therefore relied on TBS since it was a method of treatment with which they were familiar.

Patients and their families said that TBS had specialist knowledge that they had accumulated over generations. This was reinforced by the TBS they had visited who patients said had shared “success” stories with them and warned them against hospital treatment that they said was expensive, often led to amputations and meant that fractures were slow to heal. Patients and families explained that they feared hospital management and worried that they “might not make it” from surgery. They also expressed distress about the prospect of surgeons adding a foreign material or removing body parts that God had given to them (Box 4).

**Box 4: Socio-cultural or community-level factors - Illustrative quotations**

*“It is just recently that people know about health facilities treating fractures, but during our time whenever one has a fracture you are taken to the traditional bonesetter”*. Female patient, 68 years old.

*“Imagine removing what God gave you and replacing it with a metal, which was what scares me”.* Female patient, 59 years old.

#### Public policy.

During interviews, patients and caregivers discussed time to surgery. In general, those accessing private care had more timely access to surgery than those accessing care within the public system. Researchers observed that the private facilities had more resources and equipment. During one observation, a healthcare professional explained this meant patients could avoid some of the challenges in the public sector that meant some discontinued treatment, including long waits in orthopaedic clinics at public hospitals and the need to visit multiple facilities. Patients who could afford private physiotherapy explained how they arranged home visits which meant they did not have to travel to a tertiary hospital for rehabilitation, ensuring they received this care. Several patients and caregivers accessing public health facilities explained how hospital financing policies that required advance payment before treatment commenced caused delays and in many cases, led them to abandon surgical care.

## Discussion

The study explored the diverse and complex pathways that people with hip fracture follow in The Gambia from the perspective of patients and caregivers. People with fractures often moved back and forth between TBS, public health facilities, and private healthcare providers. Key factors influencing treatment-seeking were identified throughout the care pathway, across all five levels of the social ecological model [29]. At the intrapersonal level, patients often delayed seeking care because they did not think low-trauma injuries were serious. After visiting TBS, experiences of pain and dependency motivated patients to later seek biomedical care. Some older people avoided surgery because they thought they lacked the “strength” to survive an operation or “support the weight of an implant”. At the interpersonal level decisions about treatment largely rested with caregivers and was strongly influenced by financial considerations. Treatment advice from extended family and the community often favoured TBS.

Organisational factors favouring TBS included convenience and affordability. Structural challenges within the healthcare system impeded access to biomedical care and were influential in encouraging patients to visit TBS. Engagement with key components of hip fracture care was influenced by the communication between patients and caregivers with staff. This included varying information about the importance of surgery and implants and inconsistent guidance about physiotherapy. Patients and their families often felt unclear how to access hospital mobility aids. Ambiguous terminology used by providers to describe hip fractures – including breaks, cracks – influenced the perception of injury severity and therefore treatment-seeking. Socio-cultural factors influencing treatment included beliefs about the causes of fractures, traditions around visiting TBS and fears of biomedical care. At the level of public policy, healthcare financing requiring patients to pay before accessing care encouraged many to seek TBS for timely treatment.

This study’s novelty it that is has captured, for the first time, the complexity of people’s pathways through hip fracture care and the factors that shape decision-making throughout the care journey in The Gambia. Other studies conducted in Africa have examined the use of both biomedical care and traditional medicine for various health concerns, such as HIV and breast cancer [10,46]. These studies noted the drivers for patient engagement with different therapeutic modalities, rather than exploring how patients utilised both traditional and biomedical care at different points of the care journey. Previous research conducted in Africa has identified several reasons why people make use of TBS practices [47]. However, these studies have addressed how people choose between the two sectors (biomedical care and TBS), rather than making a series of decisions at different points in the care journey. Only one study carried out in Ghana characterised factors that influenced whether people chose to remain in biomedical care or revisit TBS once they had received a diagnosis [32]. As such, existing studies do not fully capture the complexity of people’s decision-making. Findings in this study have parallels with the ‘therapeutic itineraries’ mapped for other conditions including hypotensive care and tuberculosis that have shown how people utilise both traditional medicine and biomedical care during their care journeys [20,48]. These studies have highlighted how routes through care are influenced by a confluence of factors including the perceived severity of the condition [21], local beliefs about health and illness [20] and interpersonal relationships such as the influence of family members and their wider social circles [20,49]. As in our work, they also highlight the financial trade-offs made along the care pathway where resources are scarce [50,51].

Few studies have sought to develop interventions that address barriers to accessing care for musculoskeletal conditions in LMICs. However, several studies have identified opportunities for strengthening health systems to better prevent and manage musculoskeletal conditions in such settings. Briggs et al. [52] recently synthesised this research, noting the need point screening for musculoskeletal conditions to facilitate early recognition, diagnosis, and treatment, as well as the development of financing models to direct resources towards musculoskeletal health. The authors emphasise that such interventions should be contextually-adapted and go hand-in-hand with community-based education programs [52]. Our analysis identified patients’ fear and mistrust of biomedical care as a key barrier to accessing biomedical treatment for a hip fracture. Previous research conducted in high-income settings have explored patients’ distrust in biomedical care for conditions such as cancer [53]. These studies highlighted the use of provider-level interventions, such as empathetic listening, to foster trust-building within patient-provider relationships. Community-based interventions used trained community navigators to improve healthcare access [53]. There is thus a critical need for research conducted in LMIC settings to better understand public distrust of biomedical care and how this is shaped by broader socio-cultural contexts. Such research is vital for informing the development of contextually-relevant interventions in settings such as The Gambia.

The study aligns with previous research on factors influencing decision-making for musculoskeletal injuries in Africa. It highlights people’s preference for TBS treatment due to affordability [11–14,32], accessibility [12], and the ability of TBS to address both spiritual and physical causes of fractures [54,55]. High confidence in TBS has been identified across several African settings [32,47]. Fear of surgery and metal implants have also been reported in studies carried out in Ghana and Nigeria [54,56]. Similarly, the availability of effective pain relief in hospitals has been identified as an incentive for seeking biomedical care in other African countries [32]. Studies with patients in Ghana, Nigeria and Ethiopia have highlighted the strong influence of family and friends on decision-making [32,54,57,58]. People’s limited familiarity with orthopaedic services was noted as a source of anxiety and fear and made people more reluctant to seek biomedical care.

Findings in this study around the challenges in the biomedical healthcare system have been identified elsewhere [16,59]. Structural challenges within the healthcare system that were identified extend those of a recent study that assessed the availability and readiness of fracture care in The Gambia which found that only 10% of facilities had functioning X-ray machines. There were also fewer than one orthopaedic surgeon per 100,000 adults [6]. The number of trained orthopaedic nurses, occupational therapists and physiotherapists was [6]. The need for people with hip fracture to visit multiple facilities before receiving a diagnosis or operative care is partly indicative of the limited surgical capacity at District Hospitals and reliance on Tertiary Treatment Centres in many African countries [60]. The duplication of x-rays at different healthcare facilities to re-confirm a diagnosis likely reflects challenges in communication and coordination between different providers, and practices that are insufficiently flexible. This may relate to limitations in medical records in medical records infrastructure. In many settings, the absence of electronic health records, centralised record-keeping systems, and coordinated referral pathways is likely to inhibit information continuity between facilities [28].

Globally, the importance of communication between healthcare professionals and patients has been highlighted in multiple contexts. Although no studies have explored communication between healthcare professionals and patients in the delivery of orthopaedic care in Africa, the importance of effective communication during hip fracture care has been shown to be critical for optimising hip fracture outcomes in high income settings [61]. Communications should include explanations about the illness, treatment plans and options, to enable shared decision making [62]. Two recent reviews that have synthesised research on communication between patients and healthcare professionals in Southern Africa have identified similar challenges identified in this study [63,64]. Barriers to effective communication identified in these settings include high workloads [64,65], shortages of staff [64,65], busy hospital environments [66] and limited access to training to improve communication skills [65]. Frameworks to improve patient-provider communication tailored to African settings have been proposed to help address this gap [67].

Findings highlight substantial inequalities in access to surgical care based on treatment costs. In 2022, 20.3% of Gambians were estimated to be living under the international poverty line of $2.15 per day [45]. The catastrophic costs associated with treatment mean that hip fracture surgery remains unobtainable for most Gambians. Hospital financing policies that require advance payment before treatment starts also caused substantial delays while patients and their families tried to mobilise capital. Although The Gambia operates a subsidised health system, the Ministry of Health acknowledged in the National Health Policy (2021 – 2030) that patients often encounter high out-of-pocket expenditure at the point of access [28]. In 2021, the Ministry of Health enacted The National Health Insurance Bill (The National Health Insurance Bill, 2021) [68] that will establish a mandatory National Health Insurance Scheme. This will pay the healthcare costs of its members and has the potential to promote equitable access to services and reduce catastrophic healthcare expenditure [69]. However, healthcare financing is likely to remain challenging due to the low government healthcare expenditure per capita and high reliance on donor funding which is unpredictable [69]. The withdrawal of $1 million in aid by USAID that was allocated for the 2024–25 financial year will likely exacerbate this problem [70].

Given the above, patient financial constraints were identified as one of the most significant barriers to accessing hip fracture treatment, creating non-linear and fragmented care pathways. Within Africa, as in many LMICs, the high cost of surgery to patients—along with implant prices, associated expenses and travel costs — often force patients to postpone or abandon biomedical care [71]. Several strategies from different settings illustrate how these barriers can be mitigated. ‘Access now, pay later’ financing models allow patients to undergo treatment immediately and pay for it in instalments over time [71]. Similarly, recommendations from the Lancet Commission on Global Surgery advocate for standardising implant costs and negotiating bulk procurement deals between hospitals and implant manufacturers. Such agreements have the potential to make implants significantly more affordable to patients [71]. Mobile surgical clinics in Africa bring surgical services directly to rural communities, eliminating the need for costly travel to distant hospitals. These units have been shown to improve patient outcomes and increase access to emergency surgical care (21). Integrating these strategies into national health service plans and universal health coverage frameworks could help provide more equitable access to hip fracture surgery.

In the Global North, age is not a barrier to surgery for hip fracture, as the surgical procedure is designed to alleviate pain and restore mobility, even if the patient’s pre-injury mobility was limited [5]. In high-income countries, rates of non-surgical management for hip fractures are low [72,73]. This is because even in older people with limited pre-operative mobility, managing hip fractures operatively has been shown to reduce the likelihood of dying in the first year, alleviate severe pain, restore some level of mobility and prevent complications associated with prolonged immobility [5,74,75]. In The Gambia, patient age influenced decisions to operate; some older people and their caregivers said that they did not have the “strength” to survive an operation or “support the weight of an implant”. These views – held by older people and their caregivers – suggest that beliefs about older bodies’ capacity to go through and to recover from surgery have a bearing on how decisions are made about seeking or agreeing to surgery. When such views mean that access to healthcare is inequitable because of age then it may be possible to mobilise strategies to counter ageism, such as those suggested by the World Health Organization in their Global Campaign to Tackle Ageism [76]. Furthermore, older people explained that treatment decisions largely rested with caregivers. This is likely to reflect the existence of strong family ties in The Gambia where extended families are the norm and patrilineal descent systems meaning it is often the male heads of households who hold responsibility for making decisions. This aligns with findings from another study carried out in The Gambia where the eldest male in the household was often responsible for making decisions on whether to seek treatment for children with cerebral malaria [77]. The decision-making by caregivers meant that in instances when older people wanted to make choices that were different, the views of the caregivers were nonetheless paramount. An example would be older people who had asked caregivers to take them to hospital but who were taken to TBS or told to self-manage the fracture at home.

To support older people and their caregivers to make the best possible decisions about timely treatment for hip fracture, there is a need to improve public awareness of low-energy trauma and the benefits of surgical treatment [78]. Given that the greatest proportional increase in hip fracture rates of any region is predicted in Africa by 2050, this need is likely to increase [78].

### Clinical implications

The study underscores the multiple factors influencing people’s access and engagement following a fracture of the hip. It emphasises the need to co-develop, working with public, private and traditional providers, interventions that consider the complexity of people’s journeys through sectors of healthcare and the factors that shape them. Recommendations were developed from insights gained through the interviews with patients and caregivers, as well as consultations with key stakeholders and ‘experts’ in the field. As part of the analysis process outlined above, we synthesised the needs and suggestions indicated by patients and caregivers relating to hip fracture care in The Gambia and framed these within the domains of the SEM. Insights from patient and caregiver interviews were particularly valuable for formulating strategies targeting the specific needs of hip fracture patients (those relating to interpersonal, intrapersonal, and socio-cultural domains), such as the provision of health education and information. Policy- and infrastructure-related recommendations were informed by consultations with healthcare professionals involved in the delivery of hip fracture care in The Gambia, as well as by drawing on our own expertise as clinicians, gerontologists, social scientists, and health systems and policy researchers. These ongoing discussions also enabled the refinement of the recommendations devised from these patient and caregiver interviews. This multidisciplinary approach ensured that the recommendations are both practical and contextually relevant (Table 3).

**Table 3:** Recommendations for improving hip fracture care in The Gambia based on findings at the five levels of the socio-ecological model.

Recommendation		Level of the socio-ecological model				
		Intrapersonal	Interpersonal	Institutional	Socio-cultural	Policy
**Short-term**	Caregivers to be given medicine prescriptions well in advance of need to avoid caregivers searching for medications needed for immediate use, from private pharmacies every day					
	Establish hospital protocol to enable healthcare professionals to use the first set of X-rays where possible, for diagnosis, to avoid duplication					
	Provide guidance for healthcare professionals on standardized terminology to be used to describe fractures to avoid confusion amongst patients and caregivers; call a fracture a fracture.					
	Provide information to patients and caregivers on rehabilitation plans before discharge from hospital					
	Provide information to patients and caregivers on how and where to access mobility aids, with information about which aids are most appropriate					
	Deploy guidelines on time to surgery targets for hip fracture patients and regularly audit compliance					
	Increase education and awareness about the benefits of prompt surgical care for hip fractures, including the benefits for older people. Education should aim to alleviate fears around biomedical care, including correcting the belief that surgery results in amputation					
	Provide health education on operative hip fracture management for patients					
	Provide guidelines for healthcare professionals on medical optimisation of patients for surgery, outlining investigations, actions and timelines					
	Establish formal agreements between public hospitals and private implant companies to provide implants at consistent costs					
	Develop national guidelines on the management of hip fractures across The Gambia					
**Medium-term**	Improve public awareness of low-energy trauma, e.g., through public health campaigns, and school education					
	Conduct regular audits of time to surgery to identify issues that contribute to delays					
	Conduct regular audits on the availability of implants in public hospitals to manage implant procurement and availability					
	Establish an ‘access now’, ‘pay later’ model for patients and caregivers to avoid delays to emergency surgery					
	Include analgesia and osteoporosis medicines used in hip fracture care on the Essential Medicines List in The Gambia					
	Ensure mobility aids are available in hospitals, so they are accessible to patients at the point of need					
	Co-develop training programmes for TBS on fracture management to improve patient safety. This should include guidance on when to refer patients to biomedical services, e.g., in cases of hip fracture					
	Establish a professional TBS organisation within The Gambia, to register all TBS and provide a platform for engagement and training					
**Long-term**	Formally integrate TBS and biomedical care pathways, e.g., referral of patients from TBS to hospitals to access analgesia and surgery					
	Expand capacity of blood banks within The Gambia					
	Increase orthopaedic surgical capacity at district-level facilities					
	Increase availability of diagnostic radiology at public facilities					
	Establish clear referral pathways from Regional or District Hospitals to Tertiary Centres for orthopaedic surgery					
	Develop services and provide community-based physiotherapy					

### Strengths and limitations

The strengths of our ethnographic study included using case studies allowed us to explore patient and caregiver experiences within context, providing rich, in-depth information on factors impacting treatment-seeking. An iterative approach to data collection and analysis enabled us to respond to emerging insights [42]. Maximum variation sampling enabled us to identify patients from diverse hospitals, including rural, urban, and private facilities, captured a wide range of experiences [37]. Identifying patients and families through TBS allowed us to include those who had not accessed formal services, offering a fuller understanding of care pathways. This diversity was enhanced by purposive sampling of hip fracture patients [37], including those with cognitive impairment who are often excluded from studies on hip fracture care.

The study sample included more women (27 out of 38), reflecting their higher incidence of fragility fractures. A potential limitation is that some TBS were reluctant to work with researchers to recruit patients as they were concerned about them sharing information about their practices. Patients who visited these bone setters may have had different experiences of care. Nonetheless, we are confident the study provided rich information on a range of treatment-seeking behaviours. While this phase of the study captured the experiences of patients and caregivers, it did not include the perspectives of TBS or healthcare professionals involved in the organisation and delivery of hip fracture services. Including these viewpoints could have offered additional insights into patient care pathways and decision-making processes. Findings from this study are complemented by a related paper that explores TBS practices in The Gambia and their intersection with biomedical care through interviews and observations of TBS in the community [79]. We found that including hip fracture patients with cognitive impairment improved the quality of our dataset by allowing us to gather rich, first-hand experiences from this important group of patients. Nevertheless, we found that these patients had some difficulties concentrating during interviews and with memory loss when being asked to recall events. Researchers were sensitive to these difficulties during data collection, using data collection tools flexibly where patients were unable to recall past events and rephrasing questions where necessary to avoid confusion. Where patients could not recall specific events, caregivers and family members were asked to provide this information

Another strength of this study was using the SEM to inform data analysis which helped us to identify factors that impacted treatment-seeking at all five levels of the model [28]. It also enabled us to develop recommendations across multiple levels of impact [80]. To ensure data were not ‘forced’ into pre-defined constructs, data were coded inductively before being transposed onto the model. This meant that any factors that did not ‘fit’ within the model would have been identified. Themes that fitted more than one construct were discussed and collaboratively assigned to the best-fitting one. Analysis and interpretation were carried out by a research team comprised of Gambians fluent with the local language (OC, TS, KD and AT) and Zimbabwean (RC) researchers and a UK-based sociologist (SD) with experience in diverse contexts. Team members therefore were able to bring different perspectives that contributed to a deeper understanding of the issues under study. We found that using a framework approach facilitated co-working by helping the team work together to identify patterns in the data [81].

Further work is currently underway to understand how TBS interact with biomedical care from the perspective of providers and TBS. A series of engagement events have begun to explore how best to model a successful and reciprocal collaboration between TBS and biomedical services, based on learning exchange, and to determine training willingness and educational needs from both TBS and healthcare professionals. Alongside the analyses presented here, findings are being used to support the development of future programmes which will co-create equitable fracture care pathways that leverage medical pluralism to integrate TBS with healthcare professionals and enhance healthcare capacity in The Gambia. Interventions are likely to build on initiatives that have been successful in other African countries. Examples include training programmes with TBS in Ethiopia [14] and Ghana [82] and the establishment of a professional TBS organisation in Nigeria [47].

## Conclusions

This study used an ethnographic approach to map common care pathways for hip fractures in The Gambia and understand factors that impact patient access and engagement with biomedical care and traditional bone setting throughout the care pathway. Findings showed the complexity of care pathways and movement between different healthcare sectors. Using the social ecological model to inform findings highlighted the inter-related factors that influenced routes through care. Findings support the need to develop interventions that reduce the complexity of patient journeys, whilst respecting local perceptions of TBS treatment. The need to improve the integration of TBS and biomedical care is crucial in order to meet the predicted fourfold rise in hip fracture incidence in The Gambia by 2054 [83].

## Supporting information

S1 Appendix Interview topic guide. (DOCX)

S2 Appendix Observation schedule in community settings. (DOC)

S3 Appendix Observation schedule in healthcare facilities. (DOC)

S1 File Checklist. (DOCX)
