# Supplementary material for: Understanding factors that impact patient access and engagement with biomedical and traditional care for hip fractures in The Gambia: An ethnographic study using a social ecological model
Source: PLOS Glob Public Health. 2026 Jul 31;6(7):e0006626. doi: 10.1371/journal.pgph.0006626 (PMC13426917; doi:10.1371/journal.pgph.0006626)
Supplement: S1 Appendix — (DOCX) [file pgph.0006626.s001.docx]

**Fractures in Sub-Saharan Africa – The Fractures E3 Study**

**TOPIC GUIDE FOR PATIENTS AND CAREGIVERS**

Themes and subthemes explored in the interviews

**Aims and objectives**

- To understand what it is like to break a hip and their views and experiences of accessing care.

**Part 1: Find out a bit about you**

- Tell me about yourself
- Age, what you do on day-to-day basis, family, who you live with, where you live

**Part 2: Experiences of breaking hip**

- Tell me about experiences of breaking hip, what you thought had happened

**Part 3: Accessing treatment**

- Types of services visited, e.g. traditional medicine, healthcare services.
- Why?
- When you chose to get treatment, what prompted it, if delays then why?
- Aspects that made it easier/ more difficult to get treatment, e.g. cost, advice family/ friends
- Experiences/ views of treatment
- Pain
- Dignity
- Incontinence
- Disability
- Communication with clinical team

**Part 4: Recovery**

- What happened afterwards, any rehabilitation
- Changes to life

- Disability

- Productivity, e.g. work

- What they think will happen in future

- Concerns

- Impacts on life, e.g. financial implications

**Part 4: Close**

- Any other insights/ issues to discuss
