# Supplementary material for: Understanding factors that impact patient access and engagement with biomedical and traditional care for hip fractures in The Gambia: An ethnographic study using a social ecological model
Source: PLOS Glob Public Health. 2026 Jul 31;6(7):e0006626. doi: 10.1371/journal.pgph.0006626 (PMC13426917; doi:10.1371/journal.pgph.0006626)
Supplement: S2 Appendix — (DOC) [file pgph.0006626.s002.doc]

**Fractures in Sub-Saharan Africa – The Fractures E3 Study**

**OBSERVATION SCHEDULE IN COMMUNITY SETTINGS**

| **Details of observation session:**     | **Location:** |  | | --- | --- | | **Time and date:** |  | | **Observation number:** |  | | **Length of time spent observing:** |  |   **Topics to explore:**   - Description of participants, e.g. wealth, age, mobility - Description of setting, including accessibility, adaptations to environment - Activities taking place, including caring responsibilities - Interactions between family members/ people around them |  |
| --- | --- | --- | --- | --- | --- | --- | --- | --- | --- |
