# Supplementary material for: Understanding factors that impact patient access and engagement with biomedical and traditional care for hip fractures in The Gambia: An ethnographic study using a social ecological model
Source: PLOS Glob Public Health. 2026 Jul 31;6(7):e0006626. doi: 10.1371/journal.pgph.0006626 (PMC13426917; doi:10.1371/journal.pgph.0006626)
Supplement: S3 Appendix — (DOC) [file pgph.0006626.s003.doc]

**Fractures in Sub-Saharan Africa – The Fractures E3 Study**

**OBSERVATION SCHEDULE IN HEALTHCARE FACILITIES**

| **Details of observation session:**     | **Location:** |  | | --- | --- | | **Time and date:** |  | | **Observation number:** |  | | **Length of time spent observing:** |  |   **Topics to explore:**   - Types of healthcare professionals, numbers of healthcare professionals - Description of patients and carers, numbers of each - Description of setting including layout of facility - Activities taking place - Treatment protocols being followed - Interactions between professionals and patients - Interactions between professionals - Identification of barriers and facilitators to service delivery |  |
| --- | --- | --- | --- | --- | --- | --- | --- | --- | --- |
